# Supplementary material for: Deep learning based on susceptibility-weighted MR sequence for detecting cerebral microbleeds and classifying cerebral small vessel disease
Source: Biomed Eng Online. 2023 Oct 17;22:99. doi: 10.1186/s12938-023-01164-1 (PMC10580591; doi:10.1186/s12938-023-01164-1)
Supplement: Supplementary file 1 — Additional file 1: Table S1. Detailed SWS acquisition. Table S2. Results for each run of ten-fold cross-validation of the model for CSVD classification. Table S3. Results of four neurologists’ performance in classifying CSVD on the external test set. Figure S1. Confusion matrices for neurologists’ performance in classifying CSVD on the external test set. A neurologist W.L, B neurologist T.T.L, C neurologist X.X.H, D neurologist X.L. [file 12938_2023_1164_MOESM1_ESM.docx]

**Additional materials**

**Methods**

**Patients selection**

The inclusion criteria for aSVD patients were: (i) at least one arteriosclerotic risk factor, including age ≥ 55 years, hypertension, hyperglycemia, dyslipidemia; (ii) with or without CSVD symptoms, including but not limited to cognitive decline, gait and balance disturbance, parkinsonism, emotional or sleeping disorder, urinary and fecal dysfunction, or other related symptoms; (iii) with any of the following image markers on conventional MRI sequences, including T1-weighted, T2-weighted, fluid-attenuated inversion recovery (FLAIR), SWS, and diffusion-weighted imaging (DWI): white matter hyperintensities (WMHs), CMBs, enlarged perivascular spaces (EPVs), lacunes, or a combination of these image markers; (iv) no hemodynamically ≥ 50% intracranial arteriosclerotic stenosis in MR angiography; (v) without a history of large cerebral artery occlusion or cardiac embolism within 3 months prior to enrollment; (vi) without other CSVD etiologies including genetic inheritance, infection, autoimmune inflammation, neoplasm, trauma, toxication, radiation, metabolic cerebropathy, and sporadic cerebral amyloid angiopathy.

The inclusion criteria for CAA patients were: (i) age ≥ 50 years; (ii) presentation with spontaneous intracerebral haemorrhage, transient focal neurological episodes, or cognitive impairment or dementia; (iii) at least two of the following strictly lobar haemorrhagic lesions on T2-weighted MRI, in any combination: intracerebral haemorrhage, CMBs, or foci of cortical superficial siderosis or convexity subarachnoid haemorrhage; or one lobar haemorrhagic lesion plus one white matter feature (severe perivascular spaces in the centrum semiovale or white matter hyperintensities in a multispot pattern); (iv) absence of any deep haemorrhagic lesions (i.e., intracerebral haemorrhage or cerebral microbleeds) on T2-weighted MRI; (v) absence of other cause of haemorrhagic lesions, of which antecedent head trauma, haemorrhagic transformation of an ischemic stroke, arteriovenous malformation, haemorrhagic tumor, CNS vasculitis. Other causes of cortical superficial siderosis and acute convexity subarachnoid haemorrhage should also be excluded.

The inclusion criteria for CADASIL patients were: (i) age ≥ 18 years; (ii) with a confirmed genetic diagnosis of *NOTCH3* gene mutation or the presence of granular osmiophilic material on skin biopsy; (iii) a familial history of neurological disorders consistent with an autosomal dominant pattern of inheritance.

**SWS Acquisition**

**Additional file 1: Table S1.** Detailed SWS acquisition

| Dataset | MRI system | Parameters |
| --- | --- | --- |
| development set  (n= 336) | SWAN; 3.0 T GE Discovery MR750 scanner (n= 162) | field of view= 240×240; pixel size= 0.625×0.750 mm; 2 mm slice-thick; minimum repetition time; echo time= 45 ms; flip angle=15°; |
|  | SWAN; 1.5 T GE Optima MR360 scanner (n= 45) | field of view= 240×240; pixel size= 0.625×1.000 mm; 1.8 mm slice-thick; minimum repetition time; minimum echo time; flip angle=15°; |
|  | SWI; 3.0 T Siemens Prisma scanner (n= 18) | field of view= 220×220 mm; pixel size=0.700×0.700 mm; 1.5 mm slice-thick; repetition time=28 ms; echo time=20 ms; flip angle=15°; |
|  | SWAN; 1.5 T GE Healthcare Signa-creator scanner (n=34) | field of view= 240×192 mm; pixel size=0.600×1.100 mm; 2.0 mm slice-thick; repetition time=75.5 ms; echo time=49.7 ms; flip angle=20°; |
|  | SWI; 3.0 T Siemens Magnetom Verio scanner (n= 42) | field of view= 230×173mm; pixel size=1.000×0.900 mm; 2.0 mm slice-thick; repetition time=28 ms; echo time=20 ms; flip angle=15°; |
|  | SWI; 3.0 T United Imaging uMR 780 scanner (n= 35) | field of view= 230×200mm; pixel size=1.030×0.510 mm; 2.0 mm slice-thick; repetition time=30.2 ms; echo time=20 ms; flip angle=15°; |
| external test set (n= 28) | SWI; 3.0 T Siemens Magnetom Trio scanner (n= 28) | field of view= 230×230mm; pixel size=0.800×0.700mm; 2.0 mm slice-thick; repetition time=28 ms; echo time=20 ms; flip angle=15°; |

**Results**

**CSVD classification**

**Additional file 1: Table S2.** Results for each run of ten-fold cross-validation of the model for CSVD classification

|  | Internal test set | | | | External test set | | | |
| --- | --- | --- | --- | --- | --- | --- | --- | --- |
| Run | weighted-average AUC | accuracy | weighted-average precision | weighted-average F1-score | weighted-average AUC | accuracy | weighted-average precision | weighted-average F1-score |
| 1 | 0.893  (0.873-0.913) | 0.735  (0.699-0.770) | 0.667  (0.629-0.704) | 0.687  (0.648-0.726) | 0.905  (0.891-0.920) | 0.717  (0.697-0.737) | 0.569  (0.553-0.585) | 0.625  (0.609-0.642) |
| 2 | 0.783  (0.766-0.800) | 0.708  (0.662-0.753) | 0.704  (0.666-0.742) | 0.702  (0.661-0.744) | 0.904  (0.891-0.917) | 0.718  (0.681-0.754) | 0.816  (0.794-0.837) | 0.671  (0.625-0.717) |
| 3 | 0.815  (0.794-0.835) | 0.676  (0.632-0.719) | 0.722  (0.684-0.760) | 0.691  (0.651-0.731) | 0.904  (0.890-0.918) | 0.715  (0.663-0.767) | 0.763  (0.725-0.801) | 0.722  (0.674-0.771) |
| 4 | 0.834  (0.814-0.854) | 0.676  (0.635-0.716) | 0.703  (0.660-0.745) | 0.664  (0.622-0.706) | 0.909  (0.895-0.923) | 0.750  (0.708-0.791) | 0.725  (0.670-0.779) | 0.725  (0.681-0.770) |
| 5 | 0.909  (0.895-0.923) | 0.672  (0.627-0.718) | 0.815  (0.788-0.843) | 0.697  (0.655-0.740) | 0.931  (0.920-0.941) | 0.751  (0.697-0.804) | 0.760  (0.708-0.811) | 0.748  (0.694-0.802) |
| 6 | 0.841  (0.822-0.860) | 0.763  (0.732-0.793) | 0.703  (0.677-0.729) | 0.716  (0.683-0.750) | 0.928  (0.916-0.940) | 0.712  (0.675-0.749) | 0.691  (0.620-0.762) | 0.666  (0.627-0.706) |
| 7 | 0.888  (0.867-0.909) | 0.816  (0.782-0.850) | 0.819  (0.782-0.856) | 0.807  (0.770-0.844) | 0.920  (0.908-0.932) | 0.677  (0.627-0.727) | 0.750  (0.714-0.786) | 0.687  (0.639-0.736) |
| 8 | 0.808  (0.867-0.908) | 0.786  (0.746-0.825) | 0.836  (0.806-0.866) | 0.802  (0.767-0.837) | 0.918  (0.904-0.931) | 0.681  (0.626-0.736) | 0.751  (0.712-0.790) | 0.692  (0.639-0.744) |
| 9 | 0.872  (0.851-0.894) | 0.815  (0.772-0.857) | 0.856  (0.827-0.886) | 0.823  (0.783-0.863) | 0.923  (0.911-0.935) | 0.751  (0.707-0.795) | 0.741  (0.694-0.789) | 0.741  (0.695-0.788) |
| 10 | 0.820  (0.800-0.841) | 0.665  (0.620-0.709) | 0.755  (0.743-0.769) | 0.690  (0.652-0.727) | 0.917  (0.904-0.930) | 0.715  (0.668-0.762) | 0.713  (0.663-0.763) | 0.705  (0.658-0.751) |
| mean | 0.865  (0.841-0.884) | 0.732  (0.718-0.746) | 0.755  (0.743-0.768) | 0.733  (0.719-0.747) | 0.899  (0.884-0.914) | 0.717  (0.704-0.731) | 0.717  (0.702-0.733) | 0.705  (0.691-0.719) |

**Note.** the numbers in parentheses are the 95% confidence interval.

**Additional file 1: Table S3.** Results of four neurologists’ performance in classifying CSVD on the external test set

| Neurologist | Accuracy | Weighted-average precision | Weighted-average F1 score |
| --- | --- | --- | --- |
| L.W | 0.818  (0.775-0.861) | 0.834  (0.795-0.874) | 0.821  (0.779-0.863) |
| T.T.L | 0.822  (0.779-0.865) | 0.863  (0.830-0.896) | 0.830  (0.789-0.870) |
| X.H.H | 0.748  (0.694-0.803) | 0.758  (0.706-0.811) | 0.751  (0.698-0.804) |
| X.L | 0.643  (0.593-0.694) | 0.647  (0.587-0.708) | 0.628  (0.575-0.682) |

**Note.** the numbers in parentheses are the 95% confidence interval.

**
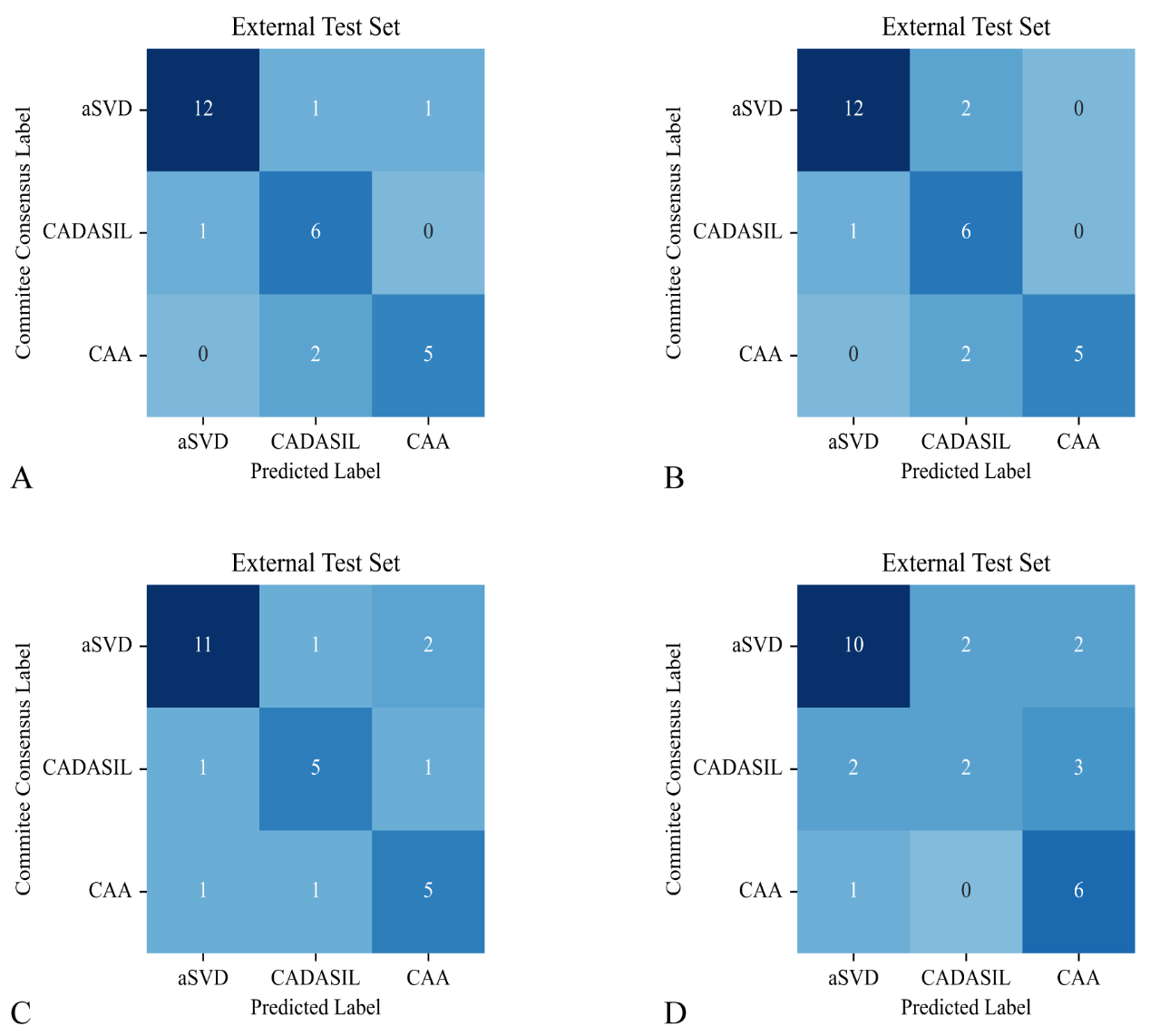
**

**Additional file 1: Figure S1.** Confusion matrices for neurologists’ performance in classifying CSVD on the external test set. (A) neurologist W.L, (B) neurologist T.T.L, (C) neurologist X.X.H, (D) neurologist X.L.
